# Effect of Adding Inhaled Corticosteroid to Long-Acting Muscarinic Antagonist/Long-Acting Beta-Agonist Therapy Among Patients With Chronic Obstructive Pulmonary Disease

**DOI:** 10.7759/cureus.19168

**Published:** 2021-10-31

**Authors:** Mizuki Yuasa, Yosuke Tanaka, Tohru Tanaka, Takeru Kashiwada, Namiko Taniuchi, Yoshinobu Saito, Masahiro Seike, Mitsunori Hino, Akihiko Gemma

**Affiliations:** 1 Department of Pulmonary Medicine and Oncology, Graduate School of Medicine, Nippon Medical School, Tokyo, JPN; 2 Respiratory Care Clinic, Nippon Medical School, Tokyo, JPN

**Keywords:** long-acting beta 2 agonist (laba), long-acting muscarinic antagonist (lama), inhaled corticosteroid (ics), airway resistance, impulse oscillation system (ios), chronic obstructive pulmonary disease

## Abstract

Background

The role of inhaled corticosteroid (ICS) in chronic obstructive pulmonary disease (COPD) is unclear. Hence, this study aimed to evaluate the efficacy of ICS as an add-on to long-acting muscarinic antagonist (LAMA)/long-acting beta 2 agonist (LABA), which was assessed using the impulse oscillation system (IOS), in patients with COPD.

Methodology

We included patients with COPD whose treatment was changed from LAMA/LABA (≥four weeks) to ICS/LAMA/LABA between April 2019 and March 2021. To gain insight into the effect and safety of ICS-containing triple therapy for COPD, pulmonary function; Short-Form 36, St. George’s Respiratory Questionnaire, COPD Assessment Test, and modified Medical Research Council scores; and airway resistance assessed using the IOS from one week before LAMA/LABA was switched to ICS/LAMA/LABA therapy until more than eight but less than twelve weeks after switching were evaluated.

Results

In total, 46 patients with COPD (mean age: 72.28 ± 7.81 years) were included in the study. None of the pulmonary function test parameters significantly changed from baseline values (mean difference in forced expiratory volume in one second [FEV1.0]: +0.032, *P* = 0.12; percentage FEV1.0 [FEV1.0%]/forced vital capacity [FVC]: −0.58, *P* = 0.42; and FVC: +0.087, *P* = 0.058). Meanwhile, the IOS showed that resonant frequency (mean difference from baseline: −2.12, *P* < 0.0001) and bodily pain scores in the St. George’s Respiratory Questionnaire (mean difference: −7.03, *P* = 0.031) significantly decreased.

Conclusions

Switching from LAMA/LABA to ICS/LAMA/LABA therapy reduces airway elasticity-to-inertial resistance ratios, which may lead to structural airway improvements in patients with COPD.

## Introduction

According to the Global Strategy for the Diagnosis, Management, and Prevention of Chronic Obstructive Pulmonary Disease (2018 Report) [1], an international guideline proposed by the Global Initiative for Chronic Obstructive Lung Disease (GOLD), chronic obstructive pulmonary disease (COPD) is a common, preventable, and treatable disease characterized by persistent respiratory symptoms and airflow limitation caused by airway and/or alveolar abnormalities commonly attributed to significant exposure to noxious particles or gases. The GOLD guideline states that chronic airflow limitation in COPD, which is caused by a combination of small airway diseases (e.g., obstructive bronchiolitis) and parenchymal destruction (emphysema), each with a relative contribution, varies among individuals. Moreover, the guideline states that chronic respiratory symptoms may precede the development of airflow limitation and may be associated with the development of acute respiratory events. Further, individuals with normal spirometry results may present with chronic respiratory symptoms, and a significant number of smokers without airflow limitation have structural evidence of lung disease characterized by the presence of emphysema, airway wall thickening, and gas trapping.

Further, the global strategy of GOLD focuses on decreasing not only forced expiratory volume in one second (FEV1) but also the COPD Assessment Test (CAT) score, which is used to evaluate the quality of life (QOL) among patients with COPD, and the modified Medical Research Council (mMRC) Dyspnea Scale score, which is utilized to determine the severity of dyspnea and the frequency of exacerbations in the year prior to assessment. Hence, therapy with long-acting muscarinic antagonist (LAMA) or long-acting beta 2 agonist (LABA) combined with inhaled corticosteroid (ICS) is recommended for all patients, except those with mild diseases who had approximately one exacerbation in the previous year [1]. In relation to this, LAMA/LABA therapy and ICS/LAMA/LABA (triple therapy) are both indicated for the treatment of COPD.

To date, with the exception of improving pulmonary function or reducing mortality in COPD, ICS has been shown to improve the QOL of patients with COPD [2]. Meanwhile, its down-titration is associated with a high FEV1 [3]; thus, the role of ICS in COPD is unclear [4-8].

By contrast, our previous retrospective study of patients with COPD who received ICS as an add-on therapy to LAMA/LABA showed significant changes in resonant frequency (Fres) based on the impulse oscillation system (IOS) [9]. The changes in Fres among patients with COPD were structural, rather than functional, as this disease is primarily characterized by expiratory dysfunction. That is, changes were likely to counteract such conditions as airway remodeling is associated with high inertial/elastic resistance ratios.

To validate the results of our previous retrospective study [9], the current study aimed to prospectively assess the efficacy and safety of four-week treatment with ICS as an add-on therapy to LAMA/LABA among patients with COPD who presented with symptoms that did not disappear completely after ≥four-week treatment with LAMA/LABA therapy alone and whose treatment with LAMA/LABA was replaced with ICS/LAMA/LABA therapy (TRELEGY ELLIPTA®100, fluticasone furoate/umeclidinium/vilanterol 100/62.5/25 µg).

## Materials and methods

Study design and participants

This was a single-center, observational, prospective study. In Japan, adding ICS to LAMA/LABA therapy is accepted for patients with COPD, except for those with asthma and COPD overlap (ACO), if their symptoms do not completely disappear after LAMA/LABA therapy.

This study was conducted between April 2019 and March 2021 to evaluate pathological changes caused by switching from LAMA/LABA to ICS/LAMA/LABA therapy in patients with COPD, except ACO, who received ≥four-week treatment with LAMA/LABA therapy and whose conditions were stable even with the presence of symptoms.

Inclusion criteria

We included the following in our study: male and female patients aged 40 years or older; those diagnosed with COPD at Nippon Medical School; those who received ≥four-week treatment with LAMA/LABA therapy; those with stable symptoms for three months that did not completely disappear after ≥four-week therapy with LAMA/LABA; those with stable COPD that was unlikely to worsen with the same treatment within three months prior to the study entry; those with COPD who were previous or current smokers; those with an FEV1/forced vital capacity (FVC) ratio of <70%; and those with any diseases associated with airflow obstruction, other than COPD, that were ruled out.

Exclusion criteria

We excluded the following patients: those with a history of asthma, bronchodilator response (BDR) to 400 mg salbutamol, as evidenced by an FEV1 change of ≥200 mL or peripheral eosinophilia (≥300 cells/µL), typical asthma symptoms of atopy, or high immunoglobulin E (IgE) levels (>170 IU/mL), and those treated with LAMA, LABA, ICS, or their combination outside of the study period.

Ethics and trial registration

The study was approved by the medical ethics committee of Nippon Medical School. All participants provided written informed consent. This study, which evaluated the efficacy of ICS as an add-on therapy to LAMA/LABA among patients with COPD, was registered in UMIN-CTR Clinical Trial (UMIN000040764) (https://upload.umin.ac.jp/cgi-open-bin/ctr_e/ctr_view.cgi?recptno=R000042394).

Study design and assessments

**Figure 1:**
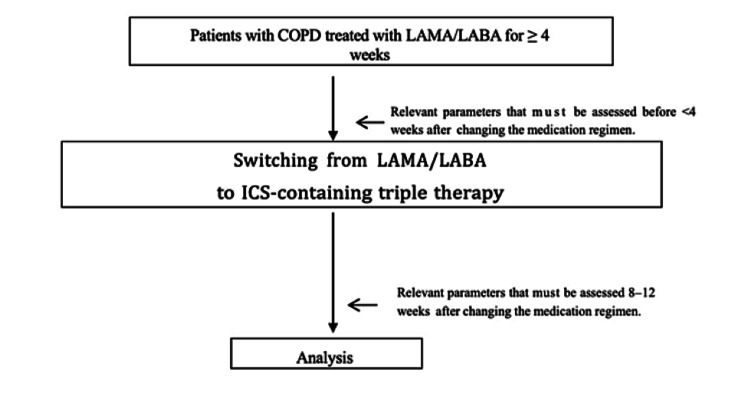
Routine COPD management flow at our hospital. We assessed for relevant parameters in all COPD patients one week before the day, when the treatment was switched to triple therapy or other drugs were used as an add-on, and more than eight but less than twelve weeks after changing their regimens, when the study drug concentration was assumed to be at the trough level. Moreover, FEV1, VC, FVC, CAT, mMRC Dyspnea Scale, SGRQ, SF-36 scores, IOS, and study drug safety were assessed. COPD: chronic obstructive pulmonary disease; ICS: inhaled corticosteroid.; LAMA: long-acting muscarinic antagonist; LABA: long-acting β-agonist.; FEV1: forced expiratory volume in one second; VC: vital capacity; FVC: forced vital capacity; CAT: COPD Assessment Test; mMRC: modified Medical Research Council; SRGQ: St. George’s Respiratory Questionnaire; SF-36: 36-Item Short-Form Health Survey; IOS: impulse oscillation system

Upon the diagnosis of COPD, all patients were assessed for BDR (as evidenced by changes in FEV1) and a documented history of asthma. The routine COPD management at our hospital involved assessing relevant parameters in all patients with COPD one week before or on the day therapies were switched or any other drugs were provided as an add-on and more than eight but less than 12 weeks after changing the regimens when the study drug concentration was assumed to be at the trough level (Table 1, Figure 1).

**Table 1:** Routine laboratory examinations. mMRC: modified Medical Research Council; CAT: COPD Assessment Test; COPD: chronic obstructive pulmonary disease

	Day of drug initiation	Treatment period (4 weeks)	
Timing	Week 0	Weeks 8–12	
			Assessment of patient characteristics	○		
Drug administration	-	○	
Assessment of subjective/objective symptoms	○	○	
Assessment of adverse reactions	-	○	
Spirometry	○	○	
Respiratory resistance	○	○	
mMRC Dyspnea Scale	○	○	
CAT	○	○	
Assessment of drug adherence	-	○	

Study endpoints

The primary endpoint was the change in airway pathology assessed using IOS, which is a method for evaluating respiratory system resistance and reactance during tidal breathing using the forced oscillation technique at weeks eight to twelve.

Impulse oscillation system

Respiratory impedance was assessed using a commercially available oscillatory system (MostGraph-22 [Rev.1.2], Chest M.I., Co. Ltd., Tokyo, Japan), which has fulﬁlled standard recommendations, as described by Shirai et al. [10,11]. The cheeks of the participants were supported while in a sitting position and wearing a nose clip. Subsequently, they were instructed to breathe quietly at the functional residual capacity level (tidal breathing) for approximately 30 seconds. The measurements were repeated until five technically acceptable records were obtained. Respiratory impedance was automatically calculated via fast Fourier transformation using a personal computer with the airflow and pressure signals in the mouths of the participants. The respiratory system resistance values at 5 and 20 Hz (R5 and R20, respectively), difference between R5 and R20 (R5-R20), respiratory system reactance at 5 Hz (X5), Fres, and low-frequency reactance area (ALX) were evaluated. Each oscillatory index was expressed as the mean value during one respiratory cycle (entire breath), the inspiratory and expiratory phases, and the difference between the inspiratory and expiratory phases.

The secondary endpoints were changes in airway pathology at weeks eight to twelve, which were evaluated using the pulmonary function tests (PFTs) (i.e., change in FEV1 on spirometry, CAT, mMRC Dyspnea Scale, St. George’s Respiratory Questionnaire (SGRQ), the MOS Short-Form 36-Item (SF-36) Health Survey scores, and drug safety.

Eligible patients receiving LAMA/LABA therapy were evaluated for all relevant parameters from one week before the day during which the treatments were switched from LAMA/LABA to triple therapy until more than eight but less than twelve weeks after switching (Table 1, Figure 1).

Study drug

The following study drugs could be prescribed at our hospital: For LAMA/LABA, ANORO ELLIPTA® - umeclidinium/vilanterol 62.5/25 µg; Spiolto® Respimat® - tiotropium/olodaterol: 2.5/2.5 µg; and ULTIBRO® Breezhaler® (110 µg of indacaterol and 63 µg of glycopyrronium bromide, which is equivalent to 50 µg of glycopyrronium). For ICS/LAMA/LABA, TRELEGY ELLIPTA® 100 - fluticasone/umeclidinium/vilanterol 100/62.5/25 µg.

Drug dosage and administration

All patients were instructed to take the drugs at the following doses: ANORO ELLIPTA® (umeclidinium/vilanterol 62.5/25 µg) one puff once daily, Spiolto® Respimat® (2.5 µg tiotropium and 2.5 µg olodaterol) two puffs once daily, ULTIBRO® Breezhaler® (110 µg of indacaterol and 63 µg of glycopyrronium bromide, which is equivalent to 50 µg of glycopyrronium) one capsule once daily, and TRELEGY ELLIPTA®100 (fluticasone furoate/umeclidinium/vilanterol 100/62.5/25 µg) one puff once daily.

Statistical analysis

Data were expressed as mean ± standard deviation (SD). Changes in individual outcome measures from baseline were compared and analyzed. Analysis of paired data was performed using the Mann-Whitney U test. Changes in trends over time were analyzed using the least-squares method. All statistical analyses were performed using JMP version 14.3 (SAS Institute Inc., Cary, NC). A two-sided *P*-value of <0.5 indicated a statistically significant difference.

## Results

Patients

In total, 46 patients with COPD who received LAMA/LABA therapy for ≥four weeks and whose treatment was switched from LAMA/LABA to triple therapy between April 2019 and March 2021 were eligible for the study. Table 2 presents the baseline characteristics of patients.

**Table 2:** Baseline characteristics of patients. FEV1: forced expiratory volume in one second; FVC: forced vital capacity; %VC: Percentage vital capacity/predicted vital capacity; CAT: COPD Assessment Test; mMRC; modified Medical Research Council

n (male)	47 (43)
Age, median (range), years	72.46 ± 7.75
Height, cm	162.9 ± 5.84
Weight, kg	57.3 ± 8.83
FEV1 (L)	1.29 ± 0.60
FEV1/FVC (%)	47.48 ± 14.84
%FEV1/predicted FEV1	57.58 ± 22.43
%VC	92.19 ± 19.62
CAT scores	14.65 ± 9.71
mMRC Dyspnea Scale scores	1.65 ± 1.34

Clinical characteristics of patients

The PFTs performed after the administration of the bronchodilator showed that the pulmonary function of all participants was within the range consistent with the diagnostic criteria for COPD according to the GOLD 2018 (Table 3). Patients with COPD who received triple therapy based on the discretion of their attending physicians were eligible for the study. Nonadherence to triple therapy was not observed during the study period.

Impulse oscillation system and pulmonary function test findings

As shown in Table 3, after switching from LAMA/LABA to triplet therapy, IOS parameters, such as R5, R20, and R5-20, all of which reflect respiratory resistance, as well as their values, which were evaluated individually in the inspiratory and expiratory phases, did not change significantly. However, the Fres significantly decreased, similar to the Fres in the expiratory/inspiratory phases (Ex Fres/In Fres). These results were consistent with those of X5 in the expiratory phase. There were no significant changes in PFT parameters (Table 3).

Symptoms and drug safety

The mMRC and CAT score did not change significantly after adding ICS to LAMA/LABA (Table 3). Moreover, although the addition of ICS was associated without significant changes in the SGRQ and SF-36 scores, body pain, which is an item in the SF-36, significantly improved. During the study period, none of the patients presented with adverse events associated with ICS-containing triple therapy. However, considering the short study duration, there were concerns regarding the safety of the proposed ICS-containing regimen.

**Table 3:** Changes in relevant parameters after switching from LAMA/LABA to ICS-containing therapy (ICS/LAMA/LABA) in patients with COPD. Data are presented as mean ± standard deviation; *: significant *P*-values. LAMA: long-acting muscarinic antagonist; LABA: long-acting beta 2 agonist; ICS: inhaled corticosteroid; COPD: chronic obstructive pulmonary disease; FEV1: forced expiratory volume in one second; FVC: forced vital capacity; %VC: percentage vital capacity/predicted vital capacity; CAT: COPD Assessment Test; mMRC: modified Medical Research Council; SGRQ: St. George’s Respiratory Questionnaire; SF-36: Short-Form 36 Health Survey; IOS: impulse oscillation system; R5: respiratory system resistance values at 5 Hz; Ex-R5: respiratory system resistance values at 5 Hz in the expiratory phase; In-R5: respiratory system resistance values at 5 Hz in the inspiratory phase; R20: respiratory system resistance values at 20 Hz; Ex-R20: respiratory system resistance values at 20 Hz in the expiratory phase; In-R20: respiratory system resistance values at 20 Hz in the inspiratory phase; X5: reactance at 5 Hz; Ex X5: reactance at 5 Hz in the expiratory phase; In X5: Reactance at 5 Hz in the inspiratory phase; Fres: frequency of resonance; Ex Fres: frequency of resonance in the expiratory phase; In Fres: frequency of resonance in the inspiratory phase

	Pre	Post	Mean difference	P-value
Pulmonary function test				
FEV1 (L)	1.29 ± 0.60	1.31 ± 0.65	+0.032	0.12
FEV1/FVC (%)	47.48 ± 14.85	46.85 ± 16.58	−0.58	0.42
%FEV1/predicted FEV1	57.58 ± 22.43	58.31 ± 23.74	+1.00	0.22
%VC	92.19 ± 19.62	92.87 ± 18.88	+1.06	0.40
FVC (L)	2.70 ± 0.75	2.77 ± 0.74	0.087	0.058
Symptoms				
CAT scores	14.65 ± 9.71	13.89 ± 9.25	−0.53	0.28
mMRC scores	1.65 ± 1.34	1.49 ± 1.18	−0.15	0.15
SGRQ scores				
Symptoms	37.98 ± 17.43	34.45 ± 17.64	−3.53	0.13
Activity	42.07 ± 22.08	40.33 ± 26.10	−1.74	0.37
Impact	21.25 ± 16.77	21.78 ± 18.24	+1.53	0.35
Total	30.76 ± 17.27	30.88 ± 19.50	+0.12	0.93
SF-36				
Physical functioning	72.39 ± 20.49	68.91 ± 20.08	−3.48	0.062
Role physical	67.93 ± 27.42	68.68 ± 27.05	+0.75	0.82
Body pain	77.91 ± 25.86	70.48 ± 28.07	−7.43	0.015*
General health	49.37 ± 12.74	46.72 ± 17.48	−2.65	0.25
Vitality	57.73 ± 22.80	62.25 ± 24.10	+4.52	0.082
Social functioning	77.55 ± 27.74	79.29 ± 24.49	+1.74	0.49
Role emotional	70.97 ± 29.88	70.92 ± 32.40	−0.048	0.99
Mental health	63.12 ± 21.54	68.15 ± 19.95	+5.028	0.053
IOS				
R5	3.39 ± 1.48	3.31 ± 1.43	−0.088	0.23
Ex-R5	3.84 ± 1.78	3.81 ± 1.72	−0.028	0.71
In-R5	2.94 ± 1.27	2.84 ± 1.22	−0.11	0.23
R20	2.55 ± 0.89	2.53 ± 1.03	−0.025	0.72
Ex-R20	2.70 ± 0.99	2.73 ± 1.16	+0.017	0.83
In-R20	2.39 ± 0.86	2.33 ± 0.95	−0.077	0.30
R5-20	0.84 ± 0.73	0.79 ± 0.61	−0.043	0.55
Ex R5-20	1.13 ± 0.94	1.08 ± 0.83	−0.052	0.45
In R5-20	0.54 ± 0.58	0.52 ± 0.54	−0.033	0.71
X5	−1.47 ± 1.21	−1.34 ± 1.07	+0.16	0.083
Ex X5	−2.01 ± 1.84	−1.72 ± 1.55	+0.31	0.034*
In X5	−0.90 ± 0.69	−0.96 ± 0.72	−0.018	0.78
Fres	15.56 ± 6.46	13.47 ± 6.02	−2.12	<0.0001*
Ex Fres	17.86 ± 8.47	15.32 ± 7.55	−2.54	<0.0001*
In Fres	13.25 ± 5.34	11.62 ± 5.36	−1.71	0.0002*

## Discussion

Several studies have shown the beneficial effects of LAMA, LABA, and LAMA/LABA therapy against airway obstruction in COPD [12-26]. This study aimed to investigate whether ICS when used as an add-on therapy to LAMA/LABA has additional benefits in COPD patients. Our results showed that none of the PFT parameters significantly changed, thereby indicating that the addition of ICS led to no direct improvement in airflow obstruction or lung volume associated with pulmonary hyperinflation in COPD. Moreover, the IOS parameters, such as R5, R20, and R5-20, did not significantly change, suggesting that the addition of ICS did not alter the location or extent of pulmonary airflow obstructive lesions in patients with COPD.

After the addition of ICS, the Fres decreased, thereby indicating a higher elastic than inertial resistance, leading to a more relaxed airway. However, there was no improvement in factors such as R5, R20, and R5-20, which are believed to be directly linked to respiratory resistance in COPD. Hence, ICS can positively affect respiratory resistance. Further, it increased elastic resistance, thereby affecting airflow in obstructive lesions, which are in the process of remodeling, and leading to a higher inertial resistance and lower elastic resistance.

Furthermore, the reason why the body pain score decreased in SGRQ after ICS addition may be related to the change in Fres after the addition of ICS; however, it could not be confirmed in this study. In previous studies, ICS, when used as an add-on therapy to inhaled bronchodilators, such as LAMA and LABA, can decrease or increase the frequency of COPD exacerbations. Hence, there is no consensus regarding the effect of ICS in patients with COPD [2-8].

In this observational study, the addition of ICS significantly decreased Fres, which was evaluated using IOS. However, it did not remarkably change pulmonary function and other related parameters. Therefore, ICS may offer an additive effect by interfering with pathological conditions in COPD, such as those characterized by airway inertia greater than elastic resistance (e.g., remodeling). ICS used as an add-on therapy to inhaled bronchodilators, such as LAMA and/or LABA, may improve Fres, an indicator that reflects structural abnormalities in the airway that develop into future airflow obstruction without changing other pulmonary function and other related parameters that affect the current symptoms and QOL. It may be one of the reasons for the accelerated FEV1 decline that occurred after the discontinuation of ICS reported by Magnussen et al. [3]. However, because this observational study included a small number of patients, it is challenging to determine whether ICS when used as an add-on therapy to LABA/LAMA can decrease the frequency of COPD exacerbations, as reported in earlier studies [4,5].

Nevertheless, our study results did not to rule out the possibility that the use of ICS may effectively decrease acute exacerbations of COPD in some patients with COPD whose pathology is characterized by airway inertia greater than elastic resistance. This finding is not consistent with that of Watz et al. Hence, the effect of ICS may be more evident in patients with COPD with significant airway inflammation, as indicated by the eosinophil count [12].

Numerous studies have shown the effect of LAMA, LABA, or LAMA/LABA on improving pulmonary function and its related mechanisms [13-29]. In contrast, very few studies have evaluated the effect ICS against COPD. This pre-post study of 46 patients who received LAMA/LABA therapy for four weeks is unlikely to provide additional insights into the efficacy of triple therapy in COPD. Hence, the changes in Fres in both the inspiratory and expiratory phases indicated that ICS might induce structural, rather than functional, changes in COPD, which is primarily characterized by expiratory dysfunction.

This study had several limitations. Most participants were men, representing the overwhelming proportion of smokers in Japan. Thus, the efficacy of ICS as an add-on therapy to LAMA/LABA in women with COPD could not be accurately identified. Moreover, this observational study included patients who had stable symptoms that did not completely disappear after treatment with LAMA/LABA for ≥four weeks, thereby indicating that their pre-ICS clinical characteristics may vary widely. LAMA/LABA therapy before switching to ICS/LAMA/LABA therapy should have been unified to ANORO ELLIPTA®, which has the same components as LAMA/LABA with TRELEGY ELLIPTA® 100. Nevertheless, the results of this study are similar to those of our previous retrospective study [9]. To validate the results of our previous retrospective study [9], the current investigation was conducted. Thus, these findings are useful for future clinical studies.

In this study, ICS-containing triple therapy did not cause major adverse events during the study period. However, considering the short study duration and the small number of participants, the safety of the proposed ICS-containing regimen remains a concern.

## Conclusions

Of the pathologies accounting for COPD, not only obstructive respiratory dysfunction but also airway remodeling responsible for permanent airflow obstruction can improve with ICS as an add-on therapy to LAMA, LABA, or LAMA/LABA. Moreover, this study showed the role of ICS as an add-on therapy in patients already receiving long-acting bronchodilators, as recommended by different guidelines. Further, the assessment of IOS in both the inspiratory and expiratory phases is useful as it can help identify a subpopulation of COPD patients who might benefit from add-on ICS therapy.
